# Reorientation of Suspended Ceramic Particles in Robocasted Green Filaments during Drying

**DOI:** 10.3390/ma15062100

**Published:** 2022-03-12

**Authors:** Bastien Dietemann, Larissa Wahl, Nahum Travitzky, Harald Kruggel-Emden, Torsten Kraft, Claas Bierwisch

**Affiliations:** 1 Fraunhofer Institute for Mechanics of Materials IWM, Wöhlerstraße 11, 79108 Freiburg, Germany; torsten.kraft@iwm.fraunhofer.de (T.K.); claas.bierwisch@iwm.fraunhofer.de (C.B.); 2 Institute of Glass and Ceramics, Friedrich-Alexander-Universität Erlangen-Nürnberg, Martensstraße 5, 91058 Erlangen, Germany; larissa.wahl@fau.de (L.W.); nahum.travitzky@fau.de (N.T.); 3 Mechanical Process Engineering and Solids Processing, TU Berlin, Ernst-Reuter-Platz 1, 10587 Berlin, Germany; kruggel-emden@tu-berlin.de

**Keywords:** evaporation, orientation prediction, particle reorientation, additive manufacturing, material extrusion

## Abstract

This work considers the fabrication of ceramic parts with the help of an additive manufacturing process, robocasting, in which a paste with suspended particles is robotically extruded. Within the final part, the material properties depend on the orientation of the particles. A prediction of the particle orientation is challenging as the part usually undergoes multiple processing steps with varying contributions to the orientation. As the main contribution to the final particle orientation arises from the extrusion process, many corresponding prediction models have been suggested. Robocasting involves, however, further processing steps that are less studied as they have a smaller influence on the orientation. One of the processing steps is drying by natural convection, which follows directly after the extrusion process. A quantification of the reorientation that occurs during drying is mostly unknown and usually neglected in the models. Therefore, we studied the amount of reorientation of suspended particles in robocasted green filaments during drying in detail. For our study, we applied the discrete element method, as it meets various requirements: The exact particle geometry can be resolved precisely; particle–particle interactions can be described; the paste composition is reproduced exactly; the initial particle orientation can be set in accordance with the prediction from the analytical models for the extrusion part; macroscopic force laws exist to represent capillary forces due to the remaining fluid phase that remains during drying. From our study, we concluded that the magnitude of particle reorientation during drying is small compared to the orientation occurring during the extrusion process itself. Consequently, reorientation during drying might further be neglected within analytical orientation prediction models.

## 1. Introduction

Robocasting, a material extrusion based additive manufacturing (EAM) technology technology, produces continuous rod-like filaments from a paste consisting of suspended microsized ceramic particles. After extrusion, robocasted filaments are subjected to at least two further processing steps, which are (i) drying by natural convection at room temperature followed by (ii) sintering. Of interest is the particle orientation within the finally processed filaments as it affects the material properties of the printed objects [1,2]. Therefore, a prediction of the particle orientation after an extrusion process is the topic of ongoing research. However, while there exist a burgeoning number of analytical orientation prediction models (OPMs) [3,4,5,6,7,8] or microscopic simulations [9,10,11] to predict such a particle orientation within the still-wet filament, little is known about the magnitude of particle reorientation in robocasted filaments during the drying step. Experimentally, information on particle reorientation is difficult to address as the particle orientation can only be measured after the sintering step, before which, however, the liquid phase must have already been removed. An experimental measurement of the orientation in the still-wet filament is hence not feasible. Instead, we quantified particle reorientation during the drying step with the help of numerical simulations.

**Figure 1:**
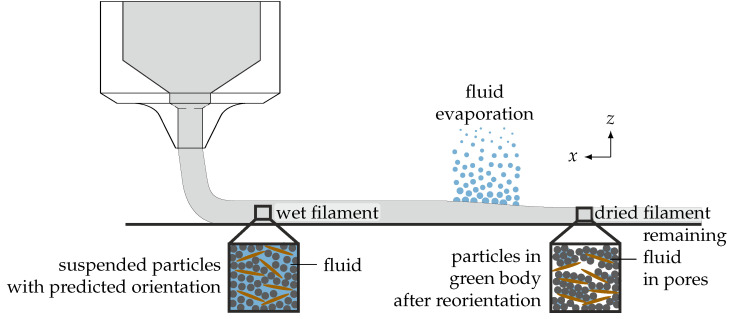
Overview figure of the scope of this work. The figure shows an illustration of the robocasting process that extrudes a ceramic filament. A zoom shows the microstructure in the wet filament that is usually predicted using numerical simulation or analytical models. After printing, the carrier fluid evaporates, during which the particle orientation changes (second microstructure), yielding the microstructure that can be measured in the experiment.

The aim of the study is visualized in Figure 1, which shows the robocasting process extruding a continuous filament. Further, a zoom on the microstructure at two different locations is shown, which should represent two different points in time: once before and once after drying. The former microstructure is the one predicted by analytical OPMs; the latter is the one measured by experiments.

**Figure 2:**
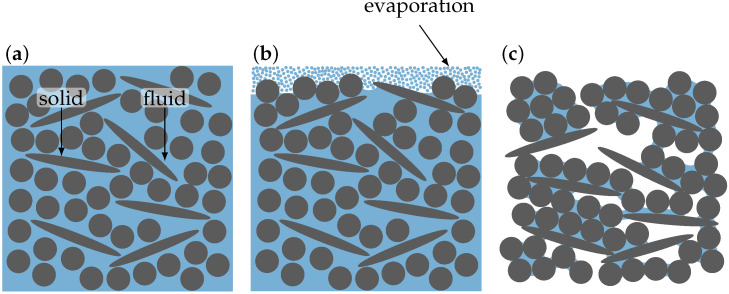
Illustration of the drying cell strategy. (**a**) shows the particle configuration in the wet filament as produced by the printing process. During the first stage of drying (**b**,**c**), most of the water evaporates and the particles change their orientation.

Drying, due to its importance for industrial applications, has been intensively studied for at least 90 y [12]. Today, it is still a practically relevant topic due to the variety of drying strategies (e.g., convection, microwaves, heat, chemical reaction, spray drying, thin layer drying, deep bed drying) and the complexity of the limiting processes therein (e.g., capillary forces, surface tension, or gradients of moisture, temperature, or pressure, all giving raise to liquid and vapor transport, as well as diffusion) [13]. Most of today’s knowledge is based on the pioneering work of Sherwood and coworkers [14,15,16,17]. The drying process is separated into at least two stages. The first stage is characterized by a constant drying rate—hence, termed the stage of constant drying rate—which depends on local conditions such as the humidity or the surrounding temperature [14,18]. During this stage, most of the particle reorientation takes place [19], and this stage was hence in the focus of our study (see Figure 2). The first stage continues while there is a continuous support of liquid molecules from the core region. Once the internal liquid transport starts to become limited, the second stage begins—also termed the stage of falling drying rate. During the second stage, the body volume stays constant and water evaporates mainly from the pores within the body’s interior. During this second stage, the internal stress state rises [20], the distribution of water varies significantly with position [21], and particle breakup is observed [22]. The consideration of the second stage was excluded from our study due to the little particle reorientation involved.

There exist at least two strategies to model drying behavior. On the one hand, several approaches are suggested to describe and to model the complex behavior taking place at the vapor–liquid interface [23,24,25,26,27,28,29,30], and they apply RVEs, as shown in Figure 2. The advantage of the RVE approach is that information on particle orientation and particle morphology can be directly imposed. However, the approach is demanding in terms of computational resources when modeling all contributing phases (solid, liquid, and vapor) and the detailed structures of the particles. Therefore, unfortunately, the RVE approach allows only studying small systems limited to a few particles [18,31,32,33,34], which are systems significantly smaller than robocasted filaments. A common solution to overcome the numerical limitations, on the other hand, is thereby to not resolve the individual particles and to rather use a continuum framework in terms of average field quantities to consider the moisture and heat transport of each phase, as well as the temperature gradients. This approach, predominantly pioneered by Luikov [35], Whitaker [36], and coworkers, allows modeling the system’s full scale [25,26,37].

Both strategies, however, suffer from limitations that make them unfeasible for the present study. A full resolution, even though such an approach has even been published by our group [30], has clearly been identified as computationally too expensive to model drying in a system of the filament’s length scale. The homogenization approach is also conceptually not applicable as the particles—whose orientation we want to study—need to be fully resolved. Instead, we applied the discrete element method (DEM), a Lagrangian particle method, that can model the effects occurring during drying. We however omitted the computation of the complex vapor–liquid interface and instead applied a virtual fluid that dries with an artificial drying rate assuming that the influence of the drying kinetics during slow drying is negligible for the final particle orientation. To represent the missing fluid phase, we applied suitable interaction laws depending on whether the particles were within the suspended or dried state. Such a strategy has also been applied elsewhere [38,39,40,41,42,43,44]; the main difference from those works is that the start configuration in this work consisted of a highly filled (3D) system of particles composed exactly as applied for the robocasting process. The initial orientation of the particles within the filament was predicted numerically taking into account the process history during robocasting [9]. Such an orientation can also be predicted with the help of analytical OPMs, which have become a standard tool within extrusion processes [45].

This paper is structured as follows. Section 2 explains the fundamentals of the numerical model. This includes the algorithm (Section 2.2), the creation of the start configuration with the according initial orientation (Section 2.4), and modeling of the drying front (Section 2.5). Section 3 follows with the results of our parameter study, which includes a comparison of the filament shrinkage measured during the experiment and simulation (Section 3.1) and the reorientation measured within our numerical parameter study (Section 3.3). Section 4 contains a discussion on the results, and Section 5 concludes this work with our suggestion about whether the effect of drying should be included within analytical orientation prediction models.

## 2. Method

### 2.1. Experimental Robocasting and Measurement of Drying Shrinkage

A robocasting paste was prepared using as-received Al_2_O_3_ powders in the shape of spherical particles (CT 3000 LS SG, Almatis, Ludwighafen, Germany, median diameter based on the volume distribution $d_{50}=0.5\;\mu m$) and platelet-like particles (RonaFlair^®^ White Sapphire, Merck Chemicals GmbH, Darmstadt, Germany, $d_{50}=9\;\mu m$). The powders were dispersed in distilled water using ammonium polymethacrylate (Darvan C-N, R.T. Vanderbilt Co., Inc., Norwalk, CT, USA) as a dispersant and mixing the dispersion using a tumbling mixer. The paste, to be suitable for printing, must have a well-defined shear-thinning behavior and yield stress. This behavior is required to allow extrusion of the paste (shear thinning) and subsequent liquid-to-solid transition for shape retention after printing (yield stress). For further details, we refer the interested reader to [46], where the measurement is explained and the corresponding flow curves are presented. In order to fabricate the paste with suitable rheological properties, an ethyl hydroxyethyl cellulose solution (Bermocoll E 320 G, Akzo Nobel GmbH, Düren, Germany) and ammonium acetate (NH_4_Ac, Merck KGaA, Darmstadt, Germany) were stepwise added as the binder and coagulant, respectively. The paste was further mixed and degassed using a planetary centrifugal mixer (ARE-250, Thinky Co., Tokyo, Japan). The solid content of the paste was set to 50 vol%, whereas this solid loading was divided into 20 vol% platelet-like powder and 30 vol% spherical powder. The paste was extruded using a Stoneflower printer (Stoneflower 3.0, Multi-Material 3D printer, Eching, Germany) equipped with a volumetric dispensing system (Vipro-HEAD 5, ViscoTec, Töging a. Inn, Germany) through nozzles with diameters of 250 μm, 580 μm and 780 μm.

**Figure 3:**
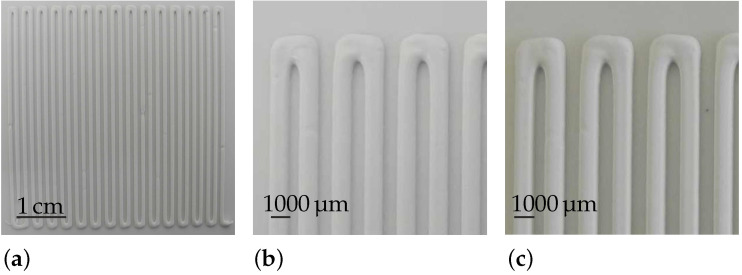
Photographs of a robocasted sample being produced with a nozzle with an 840 μm diameter. (**a**) shows the complete layer after printing; (**b**) shows a zoom into the filament; (**c**) shows the same position as in (**b**), but after drying for 24 h.

Single-layer structures were generated, and their shrinkage was determined. This was performed by taking pictures immediately after printing and after 24 h of drying at ambient conditions (Figure 3), then measuring the diameters using the image analysis software ImageJ [47].

To investigate the influence of the binder and coagulant on the drying shrinkage, additional measurements were performed with the slurry (consisting of distilled water, dispersant, and ceramic powder). For this purpose, the slurry was filled into trays and the height change during drying was investigated by means of a laser system.

### 2.2. Applying the DEM to Model Particle Reorientation during Drying

The experimental particle system is represented by an ensemble of DEM particles. Confusion might arise with respect to the terminology applied within this paper. We apply the term DEM *particle* to denote the numerical quantity of the DEM and the term *particle* to refer to the ceramic particle in the experiment.

In the DEM, we solved Newton’s equations of motion,

(1) $$\begin{array}{cc} m_{i}\;{\dot{v}}_{i} & =F_{i},\;{\dot{r}}_{i}=v_{i}\;\mathrm{and} \end{array}$$

(2) $$\begin{array}{cc} \underline{I_{i}}\;{\dot{\omega }}_{i} & =T_{i}, \end{array}$$

for an ensemble of *n* DEM particles with labels *i*, radius $R_{i}=0.25\times 10{}^{-6}$ m, mass $m_{i}=\left(4/3\right)\;\pi \;\rho_{i}\;R_{i}^{3}$, density $\rho_{i}\;=\;4\times 10{}^{3}$ kg/m${}^{3}$, center of mass positions $r_{i}$, velocities $v_{i}$, inertia tensors $\underline{I_{i}}$, angular velocities $\omega_{i}$, forces $F_{i}$, and torques $T_{i}$. The scheme was advanced in time applying a velocity Verlet scheme [48] with time step $\Delta t=\sqrt{\min\left(R_{i}\right)/\left|a_{\max}\right|}$, where $a_{\max}$ denotes the maximum acceleration of all DEM particles. We denote $r_{ij}=r_{i}-r_{j}$ as the relative position vector pointing from the position of particle *j* to particle *i* and $v_{ij}=v_{i}-v_{j}$ as the relative velocity vector, respectively. All force and torque contributions were summed up to a net resulting force:

(3) $$F_{i}=\sum_{j}\left[f_{ij}^{\;\mathrm{rep}}+f_{ij}^{\;\mathrm{visc}}+f_{ij}^{\;\mathrm{slide}}+f_{ij}^{\;\mathrm{LB}}\right]+f_{i}^{\;\mathrm{drag}}+f_{i}^{\;\mathrm{grav}}$$

and torque:

(4) $$T_{i}=\sum_{j}\left[t_{ij}^{\;\mathrm{slide}}+t_{ij}^{\;\mathrm{roll}}\right].$$

where all constituents of Equations (3) and (4) are explained in the subsequent sections. As all particles have diameters in the range of micrometers, Brownian diffusion was neglected (Péclet number Pe≫1).

#### 2.2.1. Contact Forces

DEM particles *i* and *j* in contact, i.e., $h_{ij}=R_{i}+R_{j}-\left|r_{ij}\right|>0$, were subjected to various kinds of contact forces $f_{ij}^{\mathrm{kind}}$ that were all considered by the following kinds of interactions laws. We considered elastic deformation by Hertzian repulsion reflecting the analytic solution of elastic spheres in contact [49],

(5) $$f_{ij}^{\;\mathrm{rep}}=\frac{2}{3}E_{\mathrm{eff}}\sqrt{R_{\mathrm{eff}}}\;h_{ij}^{3/2},\;f_{ij}^{\;\mathrm{rep}}=f_{ij}^{\;\mathrm{rep}}\frac{r_{ij}}{|r_{ij}|},$$

with $E_{\mathrm{eff}}=E/\left(1-\nu^{2}\right)$ using $E=1\times 10{}^{7}$ Pa as the particle’s Young’s modulus (this value was chosen to realize feasible simulation times), ν=0.25 as the particle’s Poisson’s ratio, and $R_{\mathrm{eff}}=R_{i}\;R_{j}/\left(R_{i}+R_{j}\right)$ as an effective particle radius. Dissipation of kinetic energy due to inelastic collision was considered by a viscous normal force as introduced by Landau and Lifshitz [50],

(6) $$f_{ij}^{\;\mathrm{visc}}=-\eta_{\mathrm{eff}}\;\sqrt{R_{\mathrm{eff}}\;h_{ij}}v_{ij}\;\cdot \;\frac{r_{ij}}{|r_{ij}|},\;f_{ij}^{\;\mathrm{visc}}=f_{ij}^{\;\mathrm{visc}}\frac{r_{ij}}{|r_{ij}|},$$

with $\eta_{\mathrm{eff}}=0.1\mathrm{Pa}s$ as an effective contact viscosity. We further considered two kinds of friction, which were sliding and rolling friction. Sliding friction is described by a tangential spring scheme [51] in which an imaginary spring is attached to the initial contact points when two DEM particles collide. During the finite collision time, the elongation of the spring $\xi_{ij}$ was tracked. For each particle, a force was applied at the current contact point (i.e., the center of the contact area) directed along the spring. This spring caused a force on the center of mass and a torque on the particle. For Hertzian contacts, as in our case, the approximated Hertz–Mindlin–Deresiewicz friction law, derived by DiRenzo [52], was used,

(7) $$f_{ij}^{\;\mathrm{slide}}=-\min\left[\frac{8}{3}G_{\mathrm{eff}}\sqrt{R_{\mathrm{eff}}\;h_{ij}}\;\left|\xi_{ij}\right|,\;\mu_{\mathrm{Coulomb}}\left(f_{ij}^{\;\mathrm{visc}}+f_{ij}^{\;\mathrm{rep}}\right)\right]\frac{\xi_{ij}}{|\xi_{ij}|},$$

with $G_{\mathrm{eff}}=E/\left(2\left(1+\nu \right)\right)$ as an effective shear modulus and $\mu_{\mathrm{Coulomb}}=0.2$ as a Coulomb-like friction coefficient. The corresponding sliding friction torque on particle *i* caused by particle *j* is given by:

(8) $$t_{ij}^{\mathrm{slide}}=l_{ji}\times f_{ij}^{\mathrm{slide}}$$

with the branch vector $l_{ji}$ pointing from the center of particle *i* to the contact point with particle *j*. For details of the implementation of this scheme, the reader is referred to the works of Luding [53,54]. Dissipation by rolling friction was introduced by an empirical torque on particle *i* due to particle *j* as given by Zhou [55],

(9) $$t_{ij}^{\;\mathrm{roll}}=\left\{\begin{array}{cc} -\mu_{\mathrm{roll}}\left(f_{ij}^{\;\mathrm{visc}}+f_{ij}^{\;\mathrm{rep}}\right)\frac{\omega_{i}}{|\omega_{i}|}, & \mathrm{for}\;f_{ij}^{\;\mathrm{visc}}+f_{ij}^{\;\mathrm{rep}}>0,\\ 0, & \mathrm{for}\;f_{ij}^{\;\mathrm{visc}}+f_{ij}^{\;\mathrm{rep}}\leq 0 \end{array}\right.$$

with the rolling friction coefficient $\mu_{\mathrm{roll}}=0.3\times 10{}^{-3}$ m.

#### 2.2.2. Liquid Bridge Model

During drying, water evaporates at the surface. However, not all the water content evaporates. From the experimental weight measurements, we derived that 19% of the initially provided water remained in the filament. The remaining water formed liquid bridges between particles exerting capillary forces $f_{ij}^{\;\mathrm{LB}}$ that we considered applying the liquid bridge model of Richefeu et al. [56]. This model depends on the liquid volume of the bond $V_{b}=1\times 10{}^{-20}$ m${}^{3}$ (assuming an equal distribution of liquid among all particles), the liquid–vapor surface tension $\gamma_{s}=72\;mN/m$ [57] and a particle–liquid–vapor contact angle $\theta_{s}=0{}^{\circ }$ [56,58,59]. The liquid bridge model is given by [56]:

(10) $$f_{ij}^{\;\mathrm{LB}}=\left\{\begin{array}{cc} -\kappa R, & \mathrm{for}\;\delta_{ij}<0,\\ -\kappa Re^{-\delta_{ij}/\lambda } & \mathrm{for}\;0\leq \delta_{ij}\leq d_{\mathrm{rupt}},\\ 0, & \mathrm{for}\;\delta_{ij}>d_{\mathrm{rupt}} \end{array}\right.\;f_{ij}^{\;\mathrm{LB}}=f_{ij}^{\;\mathrm{LB}}\frac{r_{ij}}{|r_{ij}|},$$

where $\delta_{ij}=-h_{ij}=\left|r_{ij}\right|-\left(R_{i}+R_{j}\right)$ denotes the separation between the DEM particles’ surfaces, $R=\sqrt{R_{i}\;R_{j}}$ is the geometrical mean radius, $\kappa =2\pi \gamma_{s}\cos\theta_{s}$ is a constant pre-factor [60], and $d_{\mathrm{rupt}}=\left(1+\theta_{s}/2\right)\sqrt[{3}]{V_{b}}$ [61] is the debonding distance above which the liquid bridge ruptures. Equation (10) includes a characteristic length λ given by [56]:

(11) $$\lambda =0.9\sqrt{\frac{V_{b}\;\left(R_{i}+R_{j}\right)}{2\;r\;R_{i}\;R_{j}}}$$

where $r=\max\{R_{i}/R_{j};R_{j}/R_{j}\}$ [56] is the maximum DEM particles’ radii ratio.

#### 2.2.3. Neighbor Independent Forces

We further considered two non-contact forces. These are gravity $f_{i}^{\;\mathrm{grav}}=m_{i}\;g$ with $g={\left(0,0,-9.81m/s^{2}\right)}^{T}$ and the Stokes drag $f_{i}^{\;\mathrm{drag}}=-6\;\pi \;\eta_{\mathrm{drag}}\;R_{i}\;v_{i}$, which is exerted onto the particle by the virtual background fluid, where $\eta_{\mathrm{drag}}$ is the background viscosity which is either $\eta_{\mathrm{air}}=2\times 10{}^{-5}$ Pa or $\eta_{\mathrm{water}}=1\times 10{}^{-3}$ m depending on whether the particle is in a dried or suspended environment.

#### 2.2.4. Discretization of Experimental Particles by Rigid Bodies

**Figure 4:**
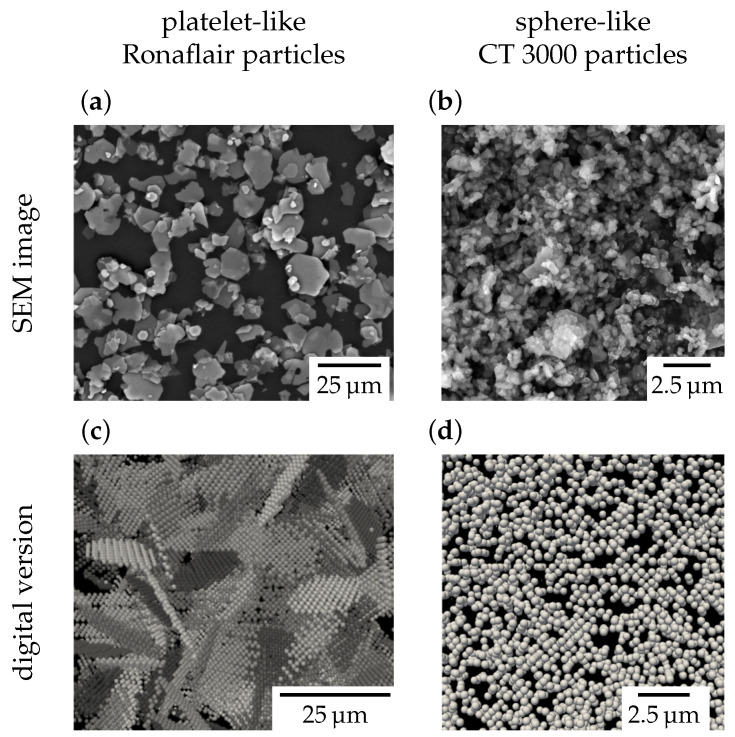
The Ronaflair (**a**) and CT 3000 particle systems (**b**) as observed by SEM images and as represented in the numerical simulation (**c**,**d**).

The correct 3D morphology of the particles was considered by a rigid body approach in which the rigid bodies are composed of a group of DEM particles with a fixed relative position. This representation, visualized in Figure 4, was applied for the platelet-like particles, but not for the sphere-like particles, which were represented by pure DEM particles.

**Figure 5:**
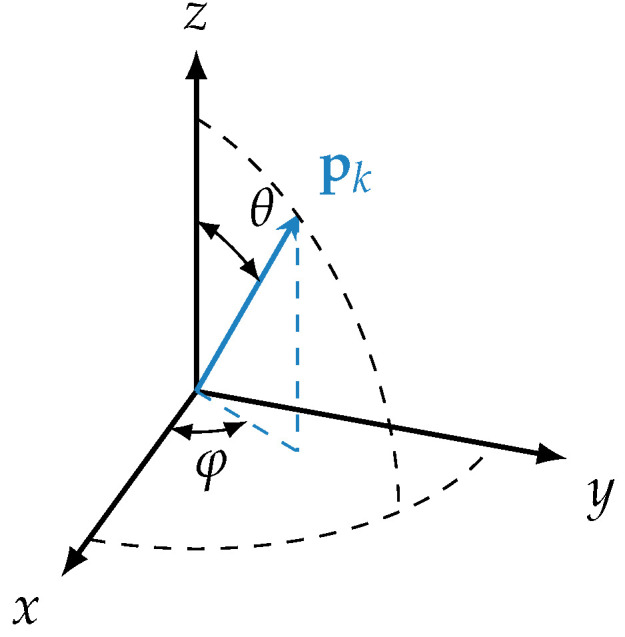
Definition of the orientation vector $p_{k}$ by spherical coordinates.

The dynamics of the rigid body motion was considered by the quaternion approach of Omelyan [62,63]. The orientation of each rigid body *k* is described by a quaternion $q_{k}={\left(\xi_{k},\eta_{k},\zeta_{k},\chi_{k}\right)}^{T}$ [62]. The quaternion $q_{k}$ is propagated in time based on acting total forces and torques acting on the rigid body. The algorithm for the implementation for time propagation was explained in detail in [64] and is therefore omitted here for the sake of brevity. While the quaternion approach yields a stable numerical tool to propagate rigid body dynamics in time, it is difficult to visualize the orientation of a quaternion $q_{k}$. A convenient illustration for orientation is its orientation vector $p_{k}$, as shown in Figure 5, which coincides with the principle axis of the rigid body.

We derived that the rigid body’s orientation vector $p_{k}$ can be computed based on the Omelyan quaternions by:

(12) $$p_{k}=\left(\begin{array}{c} -\xi_{k}^{2}+\eta_{k}^{2}-\zeta_{k}^{2}+\chi_{k}^{2}\\ 2(\zeta_{k}\chi_{k}-\xi_{k}\eta_{k})\\ 2(\eta_{k}\zeta_{k}+\xi_{k}\chi_{k}) \end{array}\right).$$

### 2.3. Mean Orientation Description by Orientation Tensors

While the orientation of each platelet is well defined in terms of the quaternion or the orientation vector, we sought for a concise description of the mean orientation of all platelets. Such a description was provided by means of a second order orientation tensor $\underline{A}$, which is defined in terms of the dyadic product of the ensemble average of all orientation vectors p, i.e., [65]:

(13) $$\underline{A}=\oint_{p}\;pp\;\psi \left(p\right)\;dp,$$

where ψ(p) is the orientation distribution function (ODE) defined on the surface of the unit sphere representing all possible values of p. From the orientation tensor, the element $A_{xx}$ describes the degree of orientation along the extrusion direction: $A_{xx}=1\left(0\right)$ means all (no) platelets are oriented along the extrusion direction. Orientation is obviously only tracked for platelets and not for spheres that have no main principle axis and therefore no orientation.

The orientation vector is usually expressed in terms of spherical coordinates, as shown in Figure 5, with a polar angle θ and an azimuthal angle φ. In this work, we mostly worked with the polar angle θ, which is used to quantify the degree of orientation within the *x*-direction as all particles are predominantly oriented within the *x*–*z*-plane. Here, $\theta =90{}^{\circ }$ refers to an alignment along the extrusion direction, i.e., the *x*-direction. Usually, the orientation tensor is favored, but for this work, we found that reorientation expressed in terms of an angle provided a more visual information than a change in the orientation tensor.

### 2.4. Start Configuration and Initial Orientation within the Wet Filament

**Figure 6:**
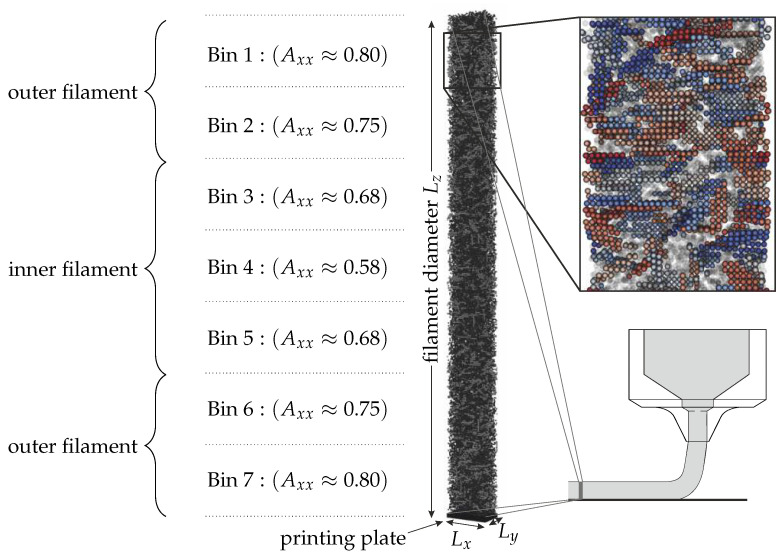
Exemplary RVEs of size $L_{z}$ = 250 μm. The figure further shows the section of the filament from which the RVEs were extracted. Additionally, a zoom into the microstructure with additional coloring of the platelet-like particle is provided. The left side of the figure shows the classification of the RVEs into the 7 bins, as well as the initial orientation in the *x*-direction and the classification into “inner” and “outer” filament regions.

The simulations were carried out in RVEs, which allowed resolving all spheres and platelets within a specific slice of the filament (see Figure 6). The filament diameter $L_{z}\in$ {250 μm, 580 μm and 780 μm} in the *z*-direction was chosen in accordance with the nozzle diameter, and the width $L_{x}=18\;\mu m$ and depth $L_{y}=25\;\mu m$ with PBC in the *x*- and *y*-direction were chosen large enough to prevent rigid body self-correlation over the PBCs and also large enough to consider a statistically relevant amount of particles (e.g., the smallest configuration with $L_{z}=250\;\mu m$ contained 120,000 DEM particles). The bottom of the RVEs was closed with a rigid wall, which represented the printing plate onto which the particles would settle during drying.

The full view in Figure 6 shows the platelets discretized by rigid bodies in light gray and the pure DEM particles to represent the spheres in dark gray. The auxiliary zoom into the filament shows the spheres as transparent to allow the inspection of the individually colored platelets. The initial orientation tensor within the filament was derived from previous works [9] in which the orientation was predicted based the process history. It could be shown that the orientation tensor varied spatially with the filament height. The filament was therefore separated into 7 compartments, each of which had its individual initial orientation tensor. We also classified the outer two bins as the “outer” filament and the inner three bins as the “inner” filament. The highest degree of alignment was at the outer layers of the filament that were close the nozzle wall during printing and the lowest degree in the nozzle center.

The DEM particle sample in the RVEs is supposed to have the same orientation as extracted from the wet filament. However, the orientation tensor of the DEM particle sample could not be set directly, and it was instead obtained by an iterative procedure. We applied an evolutionary algorithm that created a set of orientations leading to the desired orientation tensor. Afterwards, the rigid bodies were placed within the RVEs according to the pre-computed set of orientations. The remaining void spaces were filled with spheres until the desired filling fraction was reached. Placing the platelets before the spheres allowed having enough space within the RVEs so that randomly positioned platelets did not intersect with each other. Each composition was simulated with three different start configurations in order to obtain statistical averages.

### 2.5. Modeling of the Drying Process

**Figure 7:**
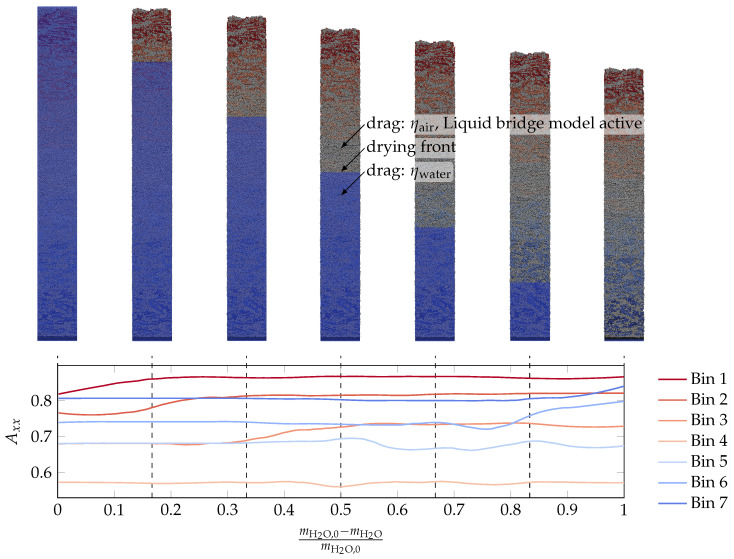
Exemplary drying simulation. The top sequence shows simulation screenshots in which the blue rectangle indicates the suspended area, while all particles above the blue rectangle are in air, but contain the remaining liquid, exerting capillary forces. The bottom graph shows the progress of the $A_{xx}$ orientation tensor element for the 7 bins that were classified in Figure 6.

Figure 7 shows an exemplary drying simulation propagating from the left to the right with the purpose to explain the concept of the numerical model. Initially, the whole filament was filled with a virtual fluid that evaporated over time. The border between the still existing virtual fluid and the already dried filament is denoted as the drying front.

This drying front propagated from the top towards the printing plate with a velocity $v_{\mathrm{df}}=500\;\mu m/s$ until it reached the printing plate. The simulation was declared as finished when DEM particle movement was no longer observed. DEM particles above the drying front were subjected to the background viscosity of air ($\eta_{\mathrm{drag}}=\eta_{\mathrm{air}}=2\times 10{}^{-5}$ Pa s). and the liquid bridge model was active; below the drying front, $\eta_{\mathrm{drag}}=\eta_{\mathrm{water}}=1\times 10{}^{-3}$ Pa s, and no liquid bridge model was active. In Figure 7, the sphere particles are shown in blue, while the platelet particles are colored according to their binning. Furthermore, the evolution of element $A_{xx}$ from the orientation tensor—which quantifies the orientation in the extrusion direction—is shown for each of the bins. The dark red particles are those close to the top part of the filament, and those particles will dry first. For these reasons, the corresponding $A_{xx}$ orientation curve changes first. Next, the drying front reaches the light red particles, which start to change their orientation. The drying front propagates further, and lastly, the dark blue particles start to be prone to drying and start to change their orientation. From each of these simulations, we compared the final orientation with the beginning of the simulation.

## 3. Results

### 3.1. Experimental and Numerical Filament Shrinkage during Drying

**Figure 8:**
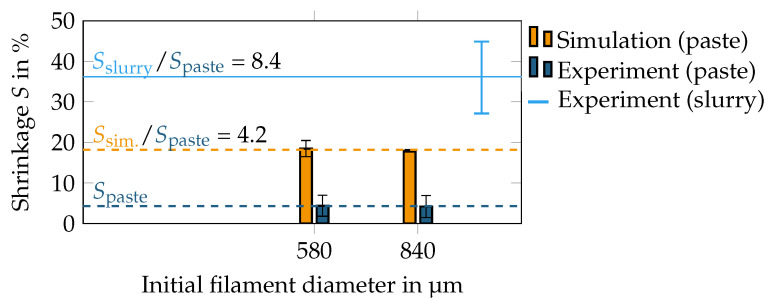
Filament shrinkage during drying as observed in the experiment and measured in the simulation. The error bars denote one standard deviation. The horizontal lines represents the mean shrinkage for a certain category.

We used the filament shrinkage during drying for a comparison between the simulation and experiment. Figure 8 shows the filament shrinkage $S=\left(L_{3}^{0}-L_{3}^{\infty }\right)/L_{3}^{0}$ within the simulation and the equivalent shrinkage of the filament diameter in the experiment, where $L_{3}^{0}$ is the initial height (or diameter) and $L_{3}^{\infty }$ the final height of the dried filament. For each diameter, three filaments were printed on each of which, 20 measurements were performed (yielding a total of 60 measurements per data point in Figure 8). As the measurement was performed after printing, any kind of swelling or elongation that might have occurred during printing was already taken into consideration. The shrinkage within the experiment was measured twice, once with the printable paste and once with a pure slurry (i.e., without the addition of the binder and coagulant). These ingredients were added to the slurry to increase the viscosity and to stabilize the green filament. Obviously, their presence affected the drying-induced shrinkage. With respect to the paste, the filament shrinkage in the simulation and in the experiment was found to be independent of the filament diameter (indicated by the dashed lines). The slurry showed substantial shrinkage around 36%. Because of this strong shrinkage, reliable measurements were only possible with a large sample volume, which was comparable to a filament diameter ≥ 1000 μm and which was not modeled numerically because of the long simulation time and the finding that the shrinkage was independent of the filament diameter. Furthermore, also the smallest diameter of 250 μm is omitted in Figure 8 as the experiment did not yield reliable data for shrinkage measurements of such a small sample. The numerical filament shrunk four-times more than the experimental filament, which can be explained by the addition of the binder, which stabilized the green filament during drying. On the other hand, the shrinkage of the slurry in the experiment was again two-times higher than predicted in the simulation, but substantially less strong than the experimental paste. The origin for the deviations in shrinkage between the experiment and simulation is further discussed in Section 4.

### 3.2. Increase of the Filling Fraction during Drying

**Figure 9:**
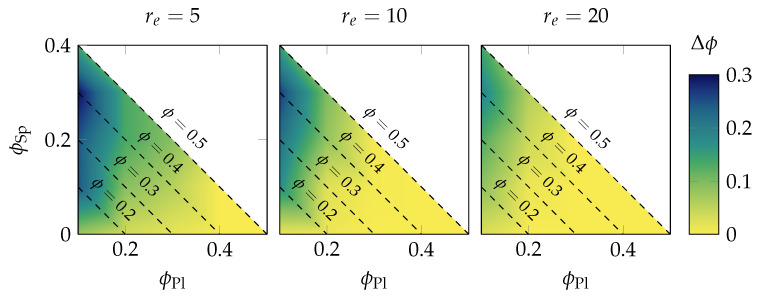
Increase in filling fraction Δϕ for various paste compositions.

Practically more interesting than the shrinkage is the final filling fraction that was realized within the filament after drying. This information is provided in Figure 9 by heat maps. Each heat map shows the increase in the filling fraction $\Delta \phi =\phi \left(t_{\infty }\right)-\phi \left(t_{0}\right)$ (beginning of simulation $t_{0}$ and end of simulation $t_{\infty }$) for a specific paste composition (the filling fraction of platelets $\phi_{\mathrm{Pl}}$ is shown on the abscissa, the filling fraction of spheres $\phi_{\mathrm{Sp}}$ on the ordinate; the dashed lines denote the isolines of the constant total filling fraction of both spheres and platelets).

Obviously, compositions with a smaller initial filling fraction have a higher potential for shrinkage compared to configurations with an initially high filling fraction. Interestingly, the change in the filling fraction did not primarily scale with the initial filling fraction, but rather with the platelets’ filling fraction. In particular, a significant increase was only found for $\phi_{\mathrm{Pl}}\leq 0.2$ as the platelets stabilized the filament otherwise and withstood the compaction. Here, the smaller the platelets’ aspect ratio, the smaller the stabilization effect, the stronger the increase of the filling fraction was. Noticeable also is the influence of the spheres as it seemed that the total maximum could be found around (0.1,0.3) and not at one of the extremes (0.1,0.0) or (0.1,0.4). The reason for this behavior is further discussed in Section 4.

### 3.3. Orientation Change during Drying

From here onwards, we focus only on the particle reorientation measured in the simulation. The mean orientation of all particles is presented in terms of the mean polar angle θ (see again Figure 5) as we found this quantity intuitively more understandable than the corresponding values of the orientation tensor. Consequently, reorientation $\Delta \theta =\theta_{\infty }-\theta_{0}$ is defined as reorientation between the end ($\theta_{\infty }$) and start of the simulation ($\theta_{0}$). This quantity Δθ is a measure of reorientation, and at the same time, it provides an interpretation about whether particles rotate towards (Δθ>0) or away from (Δθ<0) the extrusion direction.

#### 3.3.1. Influence of the Composition and Platelet Aspect Ratio

**Figure 10:**
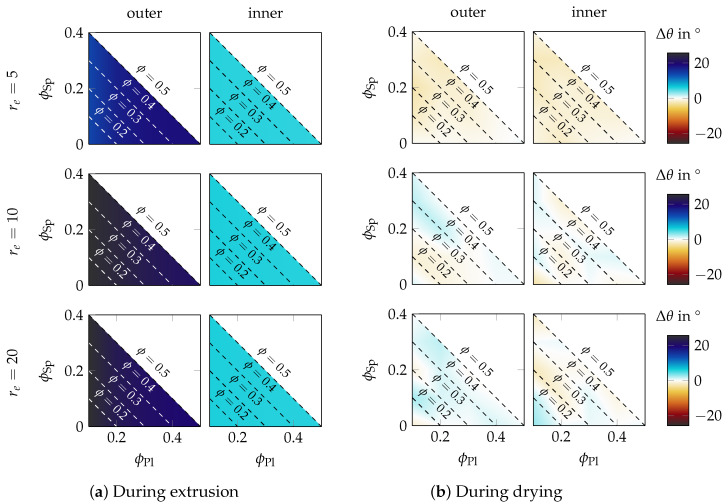
Quantification of the reorientation Δθ during drying (**b**) in direct comparison to the reorientation that occurred during the extrusion process (**a**). Each heat map shows the influence of the composition. From top to bottom, the aspect ratio $r_{e}$ of the platelets is varied.

We move on to study the amount of reorientation as a function of the composition, aspect ratio, and position within a 250 μm filament, as shown in Figure 10. This figure consists of two subfigures that are composed similarly, but show different processing steps: Subfigure (a) quantifies the reorientation that occurred during the extrusion process itself as predicted in another work [9,45]; Subfigure (b) quantifies the reorientation that occurred during drying as measured in this work. Each row represents a constant aspect ratio, which is indicated at the very left of Subfigure (a). The left column of the heat maps of each subfigure was created from all particles from the outer layer of the filament and the right column those of the inner layer (the classification into “outer” and “inner” is visualized in Figure 6). The reason for this classification was that the inner and outer particles had different initial orientations and the particles experienced a different amount of deformation during the extrusion process. Each heat map is composed similarly to those from Figure 9. The color bar on the right of Subfigure (b) applies to both subfigures, and it quantifies the reorientation of the platelets. A blue color means that particles have reoriented towards the extrusion direction, while a red color indicates a rotation in the opposite direction.

Figure 10 is the main result of this work, and therefore, it is explained more in detail. Subfigure (a), showing the reorientation during the extrusion process, generally shows a stronger reorientation compared to the drying case in Subfigure (b). This was mostly due to the large amount of shear that was acting on the outer particles during extrusion. In the filament center, elongational strain was acting on the particles, but the amount of elongation in the center was significantly smaller than the amount of shear in the outer layer. The magnitude of reorientation was therefore smaller in the inner part compared to the outer part. Obviously, reorientation depended less on the platelet’s aspect ratio or the actual composition of the paste, but rather on whether the particles were close to the outer or inner layer. In the case of drying, referring to Subfigure (b), no such process dependency existed. Here, we found a clear distinction between short platelets ($r_{e}=5$) and longer platelets ($r_{e}\geq 10$) in terms of rotation direction, and there was only a minor influence of the actual composition. Still, only a little reorientation took place during drying, which is expressed by the faint colors in Subfigure (b). The main message of Figure 10 is that the reorientation during drying was small compared to during extrusion, as revealed when directly comparing both subfigures.

#### 3.3.2. Influence of Filament Diameter

**Figure 11:**
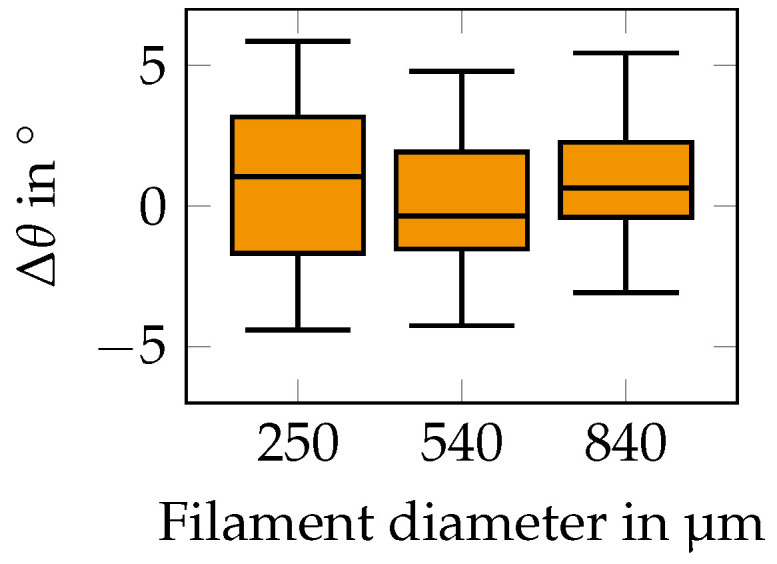
Boxplots of reorientations as observed for different initial filament diameters.

Next, we studied the reorientation as a function of the filament diameter. While previous analysis was performed for varying paste compositions, we now considered a constant paste composition of $\phi_{\mathrm{Pl}}$ = 0.2 and $\phi_{\mathrm{Sp}}$ = 0.3, which was also used in the experiment. The results are shown in the form of boxplots of the reorientation in Figure 11. A classification of the reorientation into outer and inner particles led to a similar result and is hence not shown. We found that the particles reoriented around ± 5 ${}^{\circ }$ for all filament diameters.

## 4. Discussion

### 4.1. Discussion of the Model

**Figure 12:**
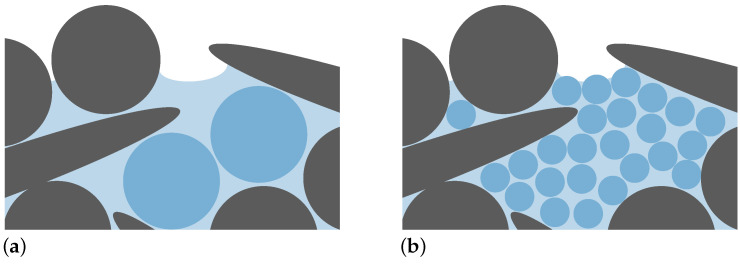
Subsection of Figure 2 close to the surface region where the fluid (light blue area) is represented by a coarse (large blue circles in in (**a**)) and by a fine (small blue circles in (**b**)) fluid resolution.

We studied the reorientation of particles within robocasted green filaments during convective drying with the help of a DEM. This model can resolve the exact geometry of the particle system and consider particle–particle interactions and the presence of remaining water by liquid bridge forces. The DEM, as a Lagrangian method, allows keeping track of the individual orientation of each ceramic particle and composing a start configuration with a defined initial orientation tensor, which we extracted from previous simulations and experimental work. However, we applied some simplifications because of the computational demands of the model. First and foremost, we neglected the experimental drying kinetics. A consideration of the correct drying kinetics requires resolving all three phases—solid, liquid, and surrounding vapor phase—as well as the thermodynamic balance between the liquid and vapor phase. Modeling of all phases, even though possible for a small number of particles [28], drastically increases the computational demand, which makes the desired simulation unfeasible within reasonable time constraints. We are not aware of any numerical simulation approach in which such a high resolution is used. This work, however, considered the whole filament diameter for a realistic extrusion nozzle size. We identified that even only resolving the fluid phase without resolving the surrounding air phase was computational too demanding. The simple reason for this claim should be explained with the help of Figure 12.

Fluid between two non-touching surface particles separated by a distance smaller than the particle discretization size will dry in reality, but not in the simulation: The length scale of the fluid discretization hence affects whether this fluid element is identified as a surface fluid particle, and hence whether it is drying (this situation is shown in Figure 12a). This situation occurred inevitably in our simulation, in which we studied high filling fractions of particles. Consequently, the drying kinetics depends on the resolution of the fluid discretization if this resolution is in the same order of magnitude as the particle sizes. This dependency is only avoided when choosing a fine fluid discretization size (Subfigure (b)). Such a resolution, however, is again not feasible with the given time constraints and the desired size of the RVEs. Instead, the current model only resolves the solid phase and considers the missing liquid phase with an effective liquid bridge model, while the underlying model parameters were extracted from experimental measurements. Still, not resolving the fluid phase also means that we did not resolve the fluid flow that was induced by particle rotation or surface tension. In this particular case, we considered omitting those effects acceptable as the dynamics of the particle system is rather determined by geometrical constraints, and, hence, we assumed that any fluid flow has a negligible influence on the final particle orientation. We also applied drying kinetics that were faster than the experimental drying kinetics. We again justified this procedure by the fact that drying happened under natural convection over 24 h, and therefore, the process was not limited by the drying kinetics. All in all, we are convinced that this model can be used to study the reorientation of particles in robocasted filaments during drying, and—with respect to the remaining models suggested in the literature—this is the best tool at hand as long as the reorientation is not accessible by experiments.

The numerical setup was motivated by the experimental paste composition and filament shape, but minor deviations existed. The experimental particles had a lognormal size distribution (more information to be found in [46]), while we applied a monodispersed size distribution within our parameter study. This distribution was chosen to better identify the influence of the aspect ratio, and the actual values of the aspect ratio were chosen with respect to the minimum and maximum occurring aspect ratios in the experimental powder. We also decided to apply RVEs with a rectangular shape instead of a circular segment, which would be a more natural choice for the circular filament. This choice was motivated by the fact that a rectangular RVE shape avoids the singularity problem at the bottom end of the segment, which would cause numerical instabilities during the simulation. In this regard, we assumed that the shape of the RVEs had an insignificant influence on the results of our parameter study.

### 4.2. Discussion of Results

The key topic of this work was a quantification of the reorientation that was studied for various paste compositions, platelet aspect ratios, and filament diameters. The important results are shown in Figure 10 and suggest that reorientation during drying is small compared to reorientation during extrusion. The reorientation was also studied as a function of filament diameter in Figure 11 for the composition applied within the experiment. In general, it was found that the filament diameter had no influence on the particle reorientation. This behavior was expected and followed the basic intuition, and we assumed it also holds for diameters outside the studied parameter range. All in all, satisfyingly, the results showed a similar trend that there was only a small amount of reorientation by ± 5 ${}^{\circ }$ throughout the whole parameter study.

The filament shrinkage during drying was compared to the experimental shrinkage for different filament diameters. We found the numerical model to overpredict the filament shrinkage compared to the experiment by a factor of four. The origin of this mismatch is unknown, but there are some possible explanations. One, in our opinion, unlikely explanation is that we underestimated the friction forces between the particles. The discretized platelets had an increased artificial surface roughness due to the spacing of the DEM particles compared to the experimental platelets. Each platelet was approximated by 120 DEM particles, a number large enough to resolve the underlying aspect ratio and geometry of the platelets, but at the same time, too small to resolve a smooth platelet surface (see again Figure 4 for the numerical representation of the platelets). A second touching platelet might become blocked within the concave region between the DEM particles. In this regard, the experimental platelets should have a smaller friction than the numerical platelets. Instead, more likely, we assumed that there existed further forces that were neglected in our model and that came from the non-volatile ingredients in the paste composition (i.e., Bermocoll) that had been added in small amounts to the paste as binder ingredients. This binder remained as a scaffold within the green filament and thereby was responsible for its stability during drying. To test this hypothesis, we reperformed the experiment again with a slurry instead of the paste (i.e., without the addition of the binder) and found the shrinkage of the slurry to be eight-times higher than the shrinkage of the paste and two-times higher than the simulation. The binder hence might have locally glued the platelets together. The consideration of the binder ingredients by a particle-based numerical model would be a whole new subject of study, and we hence preferred to neglect their influence. As we overpredicted the filament shrinkage with this choice, we might have also overpredicted the particle reorientation that had actually occurred in the experimental filament. However, the overall message of our work—reorientation can be neglected for further studies—remains true as the experimental reorientation would even be smaller than predicted in our work. This means the particle orientation after the extrusion process will largely be maintained during drying.

Shrinkage was also studied as a function of paste composition in Figure 9. This study showed that the capillary force from the liquid bridge model had a stronger influence on the drying behavior than the actual paste composition. The strength of the capillary force depends on the distance between two DEM particles: the closer the particles approach each other, the stronger the attractive force is. This fact leads to the situation that a configuration with higher initial filling fraction of spheres collapses more strongly than comparable setups with fewer spheres. The small spheres can easily occupy the void space between two platelets and thereby impose capillary forces in all directions, whereas in setups with few spheres, those spheres become simply attached to the platelets, yielding effectively even more bulky platelets. This situation holds until the filling fraction of the spheres in the initial configuration exceeds about 30 vol%, as then, the spheres lose their mobility in the dense setup, preventing them from occupying smaller void spaces. In contrast, the addition of platelets does not increase the amount of compression, as the platelets rather contribute to the isosteric hindrance in the system and thereby prevent shrinkage. All in all, it follows that the addition of small mobile particles to the system might actually increase the shrinkage during drying.

### 4.3. Limitations of This Work

The results of this work are transferable to other drying processes under the following conditions: First, we studied uncharged ceramic particles, and hence, we neglected any kind of electrostatic contributions. Depending on the exact charge, this might either increase (opposite charges) or decrease (similar charges) the friction between the particles. Second, we studied a composition of spheres and platelets in which the spheres were significantly smaller than the platelets. As such, the spheres could easily occupy the smaller void regions between the platelets, and at the same time, their mass was too small to have a significant impact on the momentum of the platelets [9]. The dynamics of a system with larger spheres might be different. Third, this work assumed that the drying kinetics can be neglected, i.e., the particle system has enough time above the drying front to move into an equilibrium state, and particle inertia has no effect. The results cannot be compared with other processes including fast drying kinetics. Fourth, the particles are micrometers in size and embedded within a system of a high filling fraction, due to which, we neglected Brownian motion. Smaller systems with nanometer-sized particles, however, are influenced by Brownian motion, and hence, both systems cannot be compared.

### 4.4. Consequences for Orientation Prediction Models and Homogenization Methods

What are the consequences of our findings for the application of analytical OPMs? Often, experimental orientation data are used to calibrate OPMs [66,67]. In an ideal case, sufficient transient data of the orientation evolution are available to enable reliable parameter fitting. Ceramic extrusion does not allow such a transient measurement because of the high filling fraction of particles and the resulting opacity of the paste. Instead of the full transient data curve, only the final experimental orientation state is used to calibrate the model parameter. Additionally, the deformation profile required for fitting must be extracted from another computational fluid dynamic (CFD) simulation, and also, the initial orientation state is unknown. This procedure assumes that the particle orientation is maintained during drying as strong reorientations during drying would severely hinder such a calibration mechanism. Successful calibrations, however, have been reported for similar systems [2,66,68]. As such, the small amount of reorientation during drying that we measured in our work agrees well with these findings. However, this statement is only true if the amount of deformation taking place during extrusion is large enough. We saw in Figure 10 that the drying-induced reorientation was small compared to the extrusion-induced reorientation for particles at the outer layer of the filament where large deformation occurred; in contrast, the drying-induced reorientation was almost found in the same order of magnitude as the extrusion-induced reorientation for particles in the center part of the filament, where only small amounts of deformation occurred. This means that drying might have a significant influence at positions where only minor deformation happens during the process.

The shrinkage might imply consequences for the application of homogenization drying methods (as introduced in Section 1). In these methods, the particles are unresolved and only considered by an average filling fraction. These methods estimate the internal flux of water and vapor based on pressure gradients and permeability (Darcy’s law [69])—the latter quantifies the easiness of flow through a medium, and consequently, it depends on the filling fraction. Usually, the focus in this kind of simulation is set on the correct prediction of the pressure gradient [34], as this is the substantial driving force, and less on the permeability. The permeability is computed once based on the initial filling fraction and then kept constant through the simulation. The experiment showed that, depending on the choice of additives, little to large shrinkage can occur during drying. Consequently, the filling fraction changes over time. As such, we suggest that for the application of the homogenization approach, the shrinkage should be determined beforehand (e.g., by experiments) to estimate the final filling fraction in order to make a wise choice for the permeability.

## 5. Conclusions

We performed DEM simulations to quantify the reorientation of particles during drying of robocasted green filaments. The filaments were composed of platelet- and sphere-like particles to represent the paste applied in the experiment. On average, particles rotated by a maximum of ± 5 ${}^{\circ }$.

Reorientation during drying is typically neglected in analytical OPMs. These are commonly applied to extrusion processes to reliably predict a mean particle orientation based on the deformation (i.e., how much shear and elongation the particles have experienced). In our opinion, the drying step should remain neglected in OPMs, because we found that reorientation during the extrusion was significantly larger than during drying. That means using only the OPM is enough for an orientation prediction as long as the particles experience a significant amount of deformation.

There are at least two open subjects that are worth addressing in future research. During the unconsidered second stage of drying, i.e., the falling rate period, the evaporation of the remaining fluid induced stress within the filament. This stress may lead to crack initiation, and hence, its avoidance is crucial for the successful establishment of ceramic additive manufacturing to guarantee a constant production quality. While there are experimental works in this direction [70,71,72], the authors are not aware of any simulation work on the particle level that studies the stress distribution in the particle network. The effect of stress can, however, not be studied with our model without discretization of the fluid phase. We assume existing models that also resolve the fluid phase are only feasible when combined with REVs that are smaller than used in this work and, thus, too small to address the research question. The second subject relates to the subsequent processing step—sintering—during which the ceramic obtains its strength. Sintering evokes a further volume reduction and thereby a potential reorientation, a quantification of which should also be performed for the present system.

## Data Availability

All the data is available within the manuscript.

## Abbreviations

The following abbreviations are used in this manuscript:

3D: three-dimensional
CFD: computational fluid dynamic
EAM: material-extrusion-based additive manufacturing
ODF: orientation distribution function
OPM: orientation prediction model
PBC: periodic boundary condition
RVE: representative volume element
SEM: scanning electron microscopy
